# Access to Education for Orphans and Vulnerable Children in Uganda: A Multi-District, Cross-Sectional Study Using Lot Quality Assurance Sampling from 2011 to 2013

**DOI:** 10.1371/journal.pone.0132905

**Published:** 2015-07-16

**Authors:** Ayobami D. Olanrewaju, Caroline Jeffery, Nadine Crossland, Joseph J. Valadez

**Affiliations:** Department of International Public Health, Monitoring and Evaluation Technical assistance and Research Group, Liverpool School of Tropical Medicine, Liverpool, United Kingdom; TNO, NETHERLANDS

## Abstract

**Objectives:**

This study estimates the proportion of Orphans and Vulnerable Children (OVC) attending school in 89 districts of Uganda from 2011 – 2013 and investigates the factors influencing OVC access to education among this population.

**Methods:**

This study used secondary survey data from OVCs aged 5 – 17 years, collected using Lot Quality Assurance Sampling in 87 Ugandan districts over a 3-year period (2011 – 2013). Estimates of OVC school attendance were determined for the yearly time periods. Logistic regression was used to investigate the factors influencing OVC access to education.

**Results:**

19,354 children aged 5-17 were included in the analysis. We estimated that 79.1% (95% CI: 78.5% – 79.7%) of OVCs attended school during the 3-year period. Logistic regression revealed the odds of attending school were lower among OVCs from Western (OR 0.88; 95% CI: 0.79 – 0.99) and Northern (OR 0.64; 95% CI: 0.56 – 0.73) regions compared to the Central region. Female OVCs had a significantly higher odds of attending school (OR 1.09; 95% CI: 1.02 – 1.17) compared to their male counterparts. When adjusting for all variables simultaneously, we found the odds of school attendance reduced by 12% between 2011 and 2012 among all OVCs (OR 0.88; 95% CI: 0.81 – 0.97).

**Conclusion:**

Our findings reinforce the need to provide continuing support to OVC in Uganda, ensuring they have the opportunity to attain an education. The data indicate important regional and gender variation that needs to be considered for support strategies and in social policy. The results suggest the need for greater local empowerment to address the needs of OVCs. We recommend further research to understand why OVC access to education and attendance varies between regions and improvement of district level mapping of OVC access to education, and further study to understand the particular factors impacting the lower school attendance of male OVCs.

## Introduction

Since the early 21^st^ century, there has been a rise in the number of orphans and vulnerable children (OVC), the majority of which live in sub-Saharan Africa [1]. Orphanhood in sub-Saharan Africa is largely attributable to the HIV/AIDS epidemic, which has left an estimated 17.3 million children orphaned due to the death of one or both parents [1, 2]. Prolonged conflict, political instability and extreme poverty in some sub-Saharan African countries also contributes to the OVC burden [3]. In 2010, an estimated 8 million of Uganda’s 17 million children were classed as OVCs—2.4 million orphans and over 5.5 million living with other vulnerabilities [4, 5].

Orphans and other vulnerable children face numerous risks resulting from the loss of protective factors such as stable home environments and economic independence [1, 6]. Risks include: depression, suicidal ideation, exploitation, stigma and poor health and educational outcomes [1, 6–16].

Despite the 2004 implementation of free universal primary education, Uganda is not on track to meet Millennium Development Goal 2- ‘primary education for all children by the year 2015’ [17]. This delay may be due in part to the high prevalence of HIV/AIDS, prolonged conflict (particularly in the northern region) and extreme poverty [18, 19]. More than other children, OVCs have repeatedly been found less likely to attend or finish school [10, 12–16, 20, 21] and those who do attend are less likely to be in the appropriate grade-for-age [9, 11, 12]. OVCs have been found to have higher dropout rates, and are more likely to perform poorly in school as factors such as stress, hunger and anxiety may well affect their ability to perform well [1, 3, 22]. Various studies in Uganda have shown an association between OVCs and higher drop-out rates and poorer school attendance when compared with age-matched peers [23, 24].

In Uganda, several key interventions are in place to meet the educational needs of OVCs. The National OVC Policy (NOP) was developed in 2004, leading to the National Strategic Program Plan for Interventions for OVCs (NSPPI) (see page 8 in [5]. Another key strategy is the Government of Uganda initiative ensuring free primary education for all children. As a result, the Forum for Education NGOs in Uganda (FENU) and UNICEF seek to improve the education of all children in Uganda with a focus on the most vulnerable children [25]. The UK Department for International Development (DFID) has committed to assist the poorest people, particularly in Northern Uganda, to alleviate their poverty and have enabled over 40,000 dropouts (mostly OVCs) to return to school [19].

Although much has been done to enable OVC access to education in Uganda, a greater understanding of the situation is required if Uganda is to meet educational attainment targets. An investigation of the OVC characteristics impacting access to education will aid in understanding which factors enable, and which impede OVCs from obtaining an education. In addition, mapping OVC access to education is essential to understand the regional differences in OVC uptake of education and to inform relevant stakeholders where best to focus attention to achieve maximum impact.

## Methods

This study uses secondary data sources collected using the Lot Quality Assurance Sampling method (LQAS), which has been used extensively to monitor and evaluate community-based programmes in Uganda and elsewhere. These data result from the roll out of LQAS as a national health sector monitoring system in Uganda, which has been supported by USAID since 2009 (http://www.starelqas.ug) [26]. Uganda used standard LQAS procedures [27, 28]. LQAS is an established analysis technique [29] originally developed as a classification method for industrial quality control during the 1920s and adapted to health sciences in the mid-1980s [27, 28, 30] to classify management units, referred to as supervision areas (SA) according to a performance target. In Uganda SA are usually counties or sub-counties. At the district level the data are aggregated as a stratified sample, which results in a prevalence estimate with a 95% confidence interval that is no greater than +/-10%. The confidence interval also narrows considerably when multiple district data are aggregated [26, 31, 32]. We refer the reader to other sources for an in-depth description of the LQAS method [29, 31, 33].

OVCs have been aptly described as “*the children who*, *in a given local setting*, *are most likely to fall through the cracks of regular programs*, *policies and traditional safety nets and therefore need to be given special attention when programs and policy are designed and implemented*.” [34].

There are different contextual definitions of OVCs that present challenges for interpreting and adapting successful OVC strategies [35]. In the Uganda data used for this analysis, the targeted OVCs were defined as children who, at the time of data collection, met at least one of the following criteria:

- orphaned by one or both parents,
- live in a household with a very sick parent/guardian for 3+ months,
- live with a critical disability/disabilities (mental or physical),
- live in a child-headed household,
- live with grandparents or caretakers aged 70 years or older.

Data collection teams comprising of health care workers were trained for LQAS data collection using the standard approach [31, 36]. Data used in this analysis were collected at three time points, 2011, 2012 and 2013, among OVCs aged 5–17 years. Education was measured from three questions:

*Are you currently in school*? *(Yes/No)*

*How many days last week did you attend school*?

*What is your highest level of education*? *(Never attended/Functional adult literacy/Incomplete primary/ O level/A level/ Post secondary/ Vocational training)*.

Three other socio-demographic variables were included in our analysis: age, sex and geographical location (district and region).

The data collected during the three different time points were merged into one dataset. All analyses were done using SPSS version 21.0. Age and district code were regarded as essential variables. A frequency analysis was used to identify outliers for age, and these were validated using birth dates. 19,354 children with reported age between 5 and 17 years were included in the final dataset for analysis. Not all Ugandan districts were surveyed at each time point, (e.g. Kampala); Table 1 shows how the data were distributed between four Uganda regions. Similar regional classifications have been used in previous studies [23, 26, 37]. The donor selected the districts surveyed each year; hence there is some variation in the geographical regions from one year to another (S1 Table).

**Table 1:** Overview of samples of OVCs included in this study from 2011–2013 in Uganda.

Year	Region	Total Number of Districts in region	Total Number ofdistricts surveyed	Sample Size (Coverage *)
**2011**	Central	24	8	770 (14.6%)
	Eastern	32	25	2471 (47.0%)
	Northern	30	2	187 (3.6%)
	Western	26	18	1832(34.8%)
	**All**	**112**	**53**	**5260 (100%)**
**2012**	Central	24	13	1337 (18.0%)
	Eastern	32	30	3035 (41.0%)
	Northern	30	7	667 (9.0%)
	Western	26	23	2370(32.0%)
	**All**	**112**	**73**	**7409 (100%)**
**2013**	Central	24	9	879 (13.1%)
	Eastern	32	25	2494 (37.3%)
	Northern	30	15	1588 (23.8%)
	Western	26	17	1724(25.8%)
	**All**	**112**	**66**	**6685 (100%)**

*Coverage: Percentage coverage of total number of OVCs sampled for each year

We used logistic regression to investigate the associations between region, time, sex and age with the outcome variable (school attendance). School attendance was defined as currently being in school at the time of the survey. First, we used the Pearson correlation coefficient to examine collinearity between any two of these variables (values above .70 are regarded as collinear). Next, we carried out a univariate logistic regression between each independent variable and school attendance. Then we considered a multivariate regression model, where all independent variables were included. All adjusted odds ratios obtained from the logistic regression models were reported with 95% Wald confidence intervals (CI).

### Ethics Statement

This study uses secondary data sources, and we have obtained permission of the Uganda Ministry of Local Government to carry out these analyses. The LQAS surveys are part of the routine district monitoring efforts, and data is collected annually. All the surveys were carried out under the auspices of the Ministry of Local Government and the guidance of the Ministry of Gender Labour and Social Development who is responsible for the oversight of OVC programmes in Uganda. The data collection teams obtain informed consent from a parent, or, if both deceased, from the guardian of the OVC.

## Results

Table 1 presents a summary of the numbers of OVCs sampled in the different regions in 2011, 2012 and 2013: 5260, 7409 and 6685, respectively. The respondents were predominantly from the Eastern region (47.0% in 2011, 41.0% in 2012 and 37.3% in 2013). Between 2011 and 2013, the proportion of respondents increased from 3.6% to 23.8% in the Northern region, and decreased from 34.8% to 25.8% in the Western region. The Central region was represented similarly over the 3 years (14.6% in 2011, 18.0% in 2012 and 13.2% in 2013).

Among all OVCs included in our study (Table 2), 52.5% of the OVCs were males while 47.5% were females. The median age of respondents was 12 years and 6.8% of OVCs sampled had completed primary education, while 9.8% of OVCs sampled had never attended school. 79.1% (CI: 78.5–79.7) of all OVCs sampled were currently enrolled in school at the time of sampling. The median number of days of school attended per week was 5 days. Females had higher rates of 5-day school attendance (79.9%; CI: 79.1–80.7) than males (78.3%; CI: 77.5–79.1).

**Table 2:** Characteristics of OVCs sampled from 2011–2013 in Uganda.

Characteristic	Number/Denominator*	Percentage
**Sex**		
Male	10125/19279	52.5%
Female	9154/19279	47.5%
**Age (5–17 years)**		
(5–8)	5249/19354	27.1%
(9–12)	6281/19354	32.5%
(13–17)	7824/19354	40.1%
Mean; Median	11.23; 12	
**Currently attends school?**		
Yes	15276/19319	79.1%
No	4043/19319	20.9%
**Attendance rates by Sex**		
Male	7913/10108	78.3%
Female	7300/9137	79.9%
**Days attended in last week**		
1	275/13705	2.0%
2	477/13705	3.5%
3	1013/13705	7.4%
4	1214/13705	8.9%
5	10726/13705	78.3%
Median	5	
**Highest level of education**		
Never attended	1872/19195	9.8%
Functional adult literacy	188/19195	1.0%
Incomplete primary	14875/19195	77.5%
Complete primary	1300/19195	6.8%
O Level	888/19195	4.6%
A Level	22/19195	0.1%
Vocational training	50/19195	0.3%

*Denominators vary due to missing data

Table 3 presents the results from the logistic regression models. The univariate regression models show that OVCs from Western (OR: 0.88; CI: 0.79–0.98) and Northern (OR 0.63; CI: 0.55–0.71) regions had lower odds of attending school relative to OVCs from the Central region. There was no significant difference between the odds of attending school for OVCs in the Eastern (OR 1.05; CI: 0.94–1.17) and Central regions. For each year rise in age, OVCs had lower odds of attending school (OR 0.98; CI: 0.97–0.99). Female OVCs had significantly higher odds of attending school (OR 1.10; CI: 1.03–1.18) than males.

**Table 3:** Odds ratios of factors influencing access of OVCs to Education in Uganda.

	Univariate Models		Multivariate model	
	Odds Ratio	95% Wald Confidence Interval	Odds Ratio	95% Wald Confidence Interval
**Region**				
Western	0.88	(0.79–0.98)*	0.88	(0.79–0.99)*
Northern	0.63	(0.55–0.71)***	0.64	(0.56–0.73)***
Eastern	1.05	(0.94–1.17)	1.05	(0.94–1.17)
Central	Ref		Ref	
**Age**	0.98	(0.97–0.99)***	0.98	(0.97–0.99)***
**Survey Year**				
2013	0.82	(0.75–0.90)***	0.90	(0.82–0.99)**
2012	0.86	(0.79–0.94)**	0.88	(0.81–0.97)**
2011	Ref		Ref	
**Sex**				
Female	1.10	(1.03–1.18)**	1.09	(1.02–1.17)*
Male	Ref		Ref	

* (p<0.05),

** (p<0.01),

*** (p<0.001)

None of the Pearson correlation coefficients were higher than .70, therefore we included all independent variables in the multivariate model (Table 3). The association results did not change when adjusting for all variables simultaneously, except for the scale of the effect of time: the odds of school attendance reduced significantly by 12.0% between 2011 and 2012 (OR 0.88; CI: 0.81–0.97), but did not vary between 2012 and 2013.

## Discussion

According to 2011 data, 91% of primary school-aged children in Uganda were enrolled in school (no data on secondary school-aged children) [38]. Our study found that only 79% of OVCs aged 5–17 years sampled between 2011 and 2013 were enrolled in school at the time of sampling, but this enrolment changed to 88% when the sample was reduced to the 10–14 years aged-group. This result suggests that OVCs in Uganda are slightly less likely to be enrolled in school than their non-orphaned, non-vulnerable peers. This lower enrolment rate corresponds with findings from other surveys conducted in Uganda and other countries within the region. Others have found that non-OVCs have higher school attendance rates than OVCs [13–16, 39]. Low enrolment and attendance may relate to lower level of academic performance exhibited by OVCs. Numerous studies indicate OVCs are less likely than their non-OVC peers to be in the appropriate grade-for-age, are less likely to enrol in secondary school and are less likely to complete age-appropriate school levels [9–12, 16, 40]. The large number of OVCs in Uganda makes ensuring enrolment in school and attainment of an education for these vulnerable youngsters crucial in the quest to achieve universal primary education (MDG 2) and in the continued development of the country [17, 41].

The Northern region of Uganda experienced the brunt of the war between the Government of Uganda and the Lord’s Resistance Army for several decades, up until the conflict’s end in 2008 [42–44]. The conflict and its aftermath have undoubtedly affected access to education for all children due to factors such as: recruitment of child soldiers, unsafe passage to educational institutions and lack of teachers [45, 46]. OVCs in this region are particularly vulnerable. Northern Uganda has twice the level of poverty as the rest of the country [19, 47] with the majority of people in the two lowest wealth quintiles residing in Northern and West Nile Regions [23]. Though our original dataset did not include information on individual household wealth, we know that OVCs in Northern Region are less likely to attend school than their peers living in other, more prosperous regions. Studies have shown economic stress has a large impact on school attendance of all children [16, 48] and that this impact may be more pronounced in the case of OVCs where a change in economic structure of the household can lead to decreased school attendance [13, 22, 49]. In some countries, findings such as these have prompted direct cash subsidies paid to caregivers of OVCs with promising results [50, 51]. Social Cash Transfers that target the “ultra-poor” households have been shown to have the most positive impact on OVC school enrolment, with increases in school enrolment by 5% and 6% demonstrated in Malawi and Uganda respectively [50]. In post-conflict areas such as Uganda’s north, UNESCO recommends that national and international communities focus on sustainable financing of education to meet the target of universal education for these very vulnerable children [46].

We found that female OVCs were significantly more likely to attend school than their male counterparts. This result contrasts with a study assessing factors associated with school attendance of orphans using DHS data from 10 sub-Saharan African countries collected between 1992 and 2000 [16]. That study found both male and female orphans had equally low school attendance rates when compared to their non-orphaned peers. There are some possible explanations for our diverging findings. One is that our research includes children with vulnerabilities other than orphanhood, whereas the 10-country study assessed only orphans. The second possibility is; the data used for our analysis was collected at least10 years after the data used in the other study, perhaps indicating a change over time in the female to male ratio of school attendance among OVCs. In keeping with our results, the 2011 Uganda DHS reports school attendance of female double orphans aged 10–14 years was higher (87.7%) than the attendance of male OVCs of the same age (80.0%) [23]. This is of particular interest because data measuring school enrolment of all children in 2010, suggests there was an equal enrolment of girls and boys in primary school (50% girl and 50%boys) and slightly higher enrolment for boys in secondary school (46% girls and 54% boys) [52]. Though *enrolment* and *attendance* should not be confused, it is interesting to note that though boys tend to be enrolled in equal or higher numbers than girls, male OVCs tend to attend school less frequently than their female counterparts. There is need for further study to understand if male OVCs are enrolled in school at the same rate as their non-OVC peers, and if so what are the particular issues that prevent them from attending school.

### Limitations

Our results show a 12% decrease in the odds of OVC school attendance between 2011 and 2012. This could be due in part to the change in proportional regional representation of OVC over the different time periods. For example, in 2011 Eastern Region made up 47% of the total respondents while Northern Region made up less than 4%. In 2012, the proportion of respondents from Eastern Region decreased to 41% of the total while respondents from Northern Region increased to 9%. The geographical areas surveyed change over the three years, with a greater overlap in the Western, Eastern and Central regions than the Northern region. This variation in proportional representation could influence the results and lead to erroneous conclusions regarding the effect of time. Further research should be done in these areas to confirm or deny these results.

The effect of potential confounding factors (e.g. socioeconomic status, distance from school, type of orphanhood [maternal, paternal or double orphan]) could not be investigated, as the data was not collected. Uganda should add these variables to the questionnaire in subsequent surveys. We recognize the constraints associated with the use of a secondary data set for analysis, and the difficulties associated with critiquing the primary data collection. We also advise caution in interpreting findings with small levels of significance due to the large data set used.

## Conclusion

The National Strategic Programme Plan for OVC Interventions was created to guide Uganda’s commitment as a country to ensure all children are able to realise their full potential, especially orphans and other vulnerable children [53]. The Ugandan Ministry of Gender, Labor and Social Development has set the target that 90% of OVCs between the ages of 10–14 years should be attending school [54]. Our results show that school enrolment of OVCs, much less attendance, is significantly lower than the 90% target, especially in certain areas of the country.

Our findings reinforce the need to provide continuing support to all orphans and vulnerable children in Uganda, with particular attention paid to male OVCs and to all those living in the Northern and Western Regions. This study also shows the important benefit of having a decentralised monitoring system to track regional differences.

## Supporting Information

S1 Table Detailed overview of samples of OVCs included in this study from 2011–2013 in Uganda. (DOCX)
