# Supplementary material for: Access to Education for Orphans and Vulnerable Children in Uganda: A Multi-District, Cross-Sectional Study Using Lot Quality Assurance Sampling from 2011 to 2013
Source: PLoS One. 2015 Jul 16;10(7):e0132905. doi: 10.1371/journal.pone.0132905 (PMC4504478; doi:10.1371/journal.pone.0132905)
Supplement: S1 Table — (DOCX) [file pone.0132905.s001.docx]

**Supporting Information**

**S1 Table. Detailed overview of samples of OVCs included in this study from 2011 – 2013 in Uganda**

| Region | District | Sample Sizes | | | |
| --- | --- | --- | --- | --- | --- |
|  |  | **2011** | **2012** | **2013** | **Total** |
| Central | Kalangala | 91 | 91 | 95 | 277 |
|  | Kayunga | 94 | 94 | 95 | 283 |
|  | Kiboga | - | 94 | 93 | 187 |
|  | Luwero | 110 | 115 | 113 | 338 |
|  | Lwengo | - | 114 | 113 | 227 |
|  | Masaka | 112 | 113 | - | 225 |
|  | Mityana | 94 | 91 | 95 | 280 |
|  | Mpigi | 89 | 94 | 93 | 276 |
|  | Mubende | - | 131 | 0 | 131 |
|  | Mukono | - | 129 | 0 | 129 |
|  | Nakasongola | 87 | 90 | 87 | 264 |
|  | Rakai | - | 91 | - | 91 |
|  | Ssembabule | 93 | 90 | 95 | 278 |
|  | **Total** | **770** | **1337** | **879** | **2986** |
| Eastern | Amuria | - | 113 | 114 | 227 |
|  | Budaka | 95 | 95 | 92 | 282 |
|  | Bududa | 131 | 132 | 129 | 392 |
|  | Bugiri | 89 | 90 | 95 | 274 |
|  | Bukedea | - | 94 | - | 94 |
|  | Bukwa | 93 | 93 | 93 | 279 |
|  | Bulambuli | 91 | 93 | 95 | 279 |
|  | Busia | 92 | 95 | 94 | 281 |
|  | Butaleja | 110 | 109 | 111 | 330 |
|  | Buyende | 95 | 90 | 95 | 280 |
|  | Iganga | 86 | 84 | 92 | 262 |
|  | Jinja | 130 | 133 | - | 263 |
|  | Kaberamaido | - | 92 | - | 92 |
|  | Kaliro | 87 | 88 | 89 | 264 |
|  | Kamuli | 94 | 92 | 92 | 278 |
|  | Kapchorwa | 89 | 95 | 91 | 275 |
|  | Katakwi | - | 131 | 132 | 263 |
|  | Kibuku | 94 | 94 | 93 | 281 |
|  | Kumi | 108 | 111 | 107 | 326 |
|  | Kween | 90 | 93 | 94 | 277 |
|  | Luuka | 89 | 85 | 86 | 260 |
|  | Manafwa | 111 | 113 | - | 224 |
|  | Mayuge | 93 | 88 | 91 | 272 |
|  | Mbale | 128 | 125 | 128 | 381 |
|  | Namayingo | 90 | 88 | 81 | 259 |
|  | Namutumba | 90 | 94 | 89 | 273 |
|  | Pallisa | 92 | 89 | 95 | 276 |
|  | Sironko | 91 | 93 | 89 | 273 |
|  | Soroti | - | 131 | 127 | 258 |
|  | Tororo | 113 | 112 | - | 225 |
|  | **Total** | **2471** | **3035** | **2494** | **8000** |
| Northern | Adjumani | - | 93 | - | 93 |
|  | Agago | - | - | 112 | 112 |
|  | Alebatong | - | - | 93 | 93 |
|  | Amolatar | - | - | 113 | 113 |
|  | Amuru | - | - | 93 | 93 |
|  | Apac | - | 113 | 114 | 227 |
|  | Arua | 94 | 93 | - | 187 |
|  | Dokolo | - | - | 113 | 113 |
|  | Gulu | - | - | 109 | 109 |
|  | Kitgum | - | - | 94 | 94 |
|  | Koboko | - | 94 | - | 94 |
|  | Kole | - | - | 92 | 92 |
|  | Kotido | - | 89 | - | 89 |
|  | Lamwo | - | - | 111 | 111 |
|  | Lira | - | - | 114 | 114 |
|  | Nakapiripirit | - | 91 | - | 91 |
|  | Nebbi | 93 | 94 | - | 187 |
|  | Nwoya | - | - | 96 | 96 |
|  | Otuke | - | - | 109 | 109 |
|  | Oyam | - | - | 132 | 132 |
|  | Pader | - | - | 93 | 93 |
|  | Adjumani | - | 93 | - | 93 |
|  | **Total** | **187** | **667** | **1588** | **2442** |
| Western | Buhweju | 95 | 96 | 96 | 287 |
|  | Buliisa | - | 95 | - | 95 |
|  | Bundibugyo | - | 90 | - | 90 |
|  | Bushenyi | 94 | 95 | 95 | 284 |
|  | Hoima | - | 106 | - | 106 |
|  | Ibanda | 112 | 114 | 110 | 336 |
|  | Isingiro | 92 | 95 | 92 | 279 |
|  | Kabale | 128 | 130 | 128 | 386 |
|  | Kabarole | 112 | 113 | - | 225 |
|  | Kamwenge | 94 | 91 | 94 | 279 |
|  | Kanungu | 95 | 94 | 91 | 280 |
|  | Kasese | 94 | 92 | 94 | 280 |
|  | Kibaale | - | 128 | 129 | 257 |
|  | Kiruhura | 94 | 92 | 93 | 279 |
|  | Kisoro | 114 | 113 | 110 | 337 |
|  | Kyenjojo | 110 | 112 | 102 | 324 |
|  | Masindi | - | 112 | - | 112 |
|  | Mbarara | 111 | 114 | - | 225 |
|  | Mitoma | 95 | 94 | 95 | 283 |
|  | Ntungamo | 113 | 112 | 114 | 339 |
|  | Rubirizi | 93 | 96 | 94 | 283 |
|  | Rukungiri | 95 | 93 | 94 | 282 |
|  | Sheema | 91 | 93 | 93 | 277 |
|  | **Total** | **1832** | **2370** | **1724** | **5925** |
